# Appearance of the Tongue in Disease

**Published:** 1879-11

**Authors:** John A. Henning

**Affiliations:** Red Key, Ind.


### Selections.

### APPEARANCE OF THE TONGUE IN DISEASE.

BY JOHN A. HENNING, M. D., OF RED KEY, IND.

1. The elongated and pointed tongue invariably indicates irritation and determination of blood to the stomach and intestines. The extremities are often cold. It is also associated with excitation of the nerve centres.

This tongue is often found, but more especially among children. The indications are to allay irritation and divert the blood from the stomach and bowels. We should be very careful how we make our prescription in such cases, as, if we give an irritant cathartic it invariably aggravates the disease.

2. The pinched and shrunken tongue indicates atony of the digestive organs, often found in dyspepsia and kindred diseases. The treatment is plain, the pathological conditions being evident at a glance, from the appearance of the tongue.

3. The coating (saburra) or fur should be well studied. It may be greater or less in thickness, dry or moist, or clammy, more accumulated at the posterior portion.

It is said that when the tongue is heavily coated at the base with a deep yellow coat, the liver is at fault. This is not always the case; and from my observation, more often not the case. I have seen cases of jaundice with a white-coated tongue. Tobacco chewers nearly always have a yellow-coated tongue, and their liver may be sound.

4. The dry tongue has a very important significance. When we have patients who are suffering from some form of fever, pneumonia, or any other acute disease, with such a tongue, they are in danger, and require close attention. In such cases nutrition and assimilation are suspended, and food can not well be taken, and if taken can not be properly assimilated. When given, it should be in fluid form, and always above the temperature of 100°, and of a character nutritive and digestible. The digestive organs can do but little work, yet proper food given at proper intervals does good; but these organs need all the rest they can get until the disease is subdued.

Dryness of the tongue is also associated with vascular excitement, and particularly with excitation of the ganglionic and nerve centres. Hence the arrest of secretion and this dryness. Here we readily read the state of the nervous system.

In many cases the sympathetic nerve is not only excited and irritated, but there is involuntary contraction of muscular tissue, thus suspending the secretions of the several organs.

The indications are proper sedatives for the vascular excitement, and diaphoretics for contractions or excitement of the nerves, associated with other proper treatment. By this course we shall soon see our patient with a moist tongue, and some of the secretions re-established.

5. Often the tongue changes in the disease, from the dryness above referred to to a brown or black color, with sordes about the teeth. The common idea is that the system is in a typhoid condition. This is true, yet it undoubtedly means, also, that the blood is in a septic condition; a very important fact for us to know. Then our best antiseptics should be given, with stimulants and tonics.

Thus we can readily read, from the appearance of the tongue, the condition of the digestive organs, function of nutrition and assimilation, the condition of the nervous system and the state of the blood. Of course, we must take all other symptoms into consideration, yet the appearances of the tongue, as pointed out, seldom fail in giving us, at a glance, valuable information as to the true condition of the system.

